# Otomicroscopic and histologic findings of induced myringosclerosis in rats: a critical study of an experimental model

**DOI:** 10.1016/S1808-8694(15)31272-6

**Published:** 2015-10-20

**Authors:** Patrícia F. Santos, Mariana C. Leal, Cristina Peixoto, Silvio Caldas Neto, Silvania Tavares Paz Rosas

**Affiliations:** 1 Master in Surgery, Federal University of Pernambuco.; 2 Post-graduation in Otorhinolaryngology under course, University of Sao Paulo. Deputy Professor, Department of Surgery, Federal University of Pernambuco.; 3 Ph.D., Joint Professor, Department of Molecular Biology, Centro de Pesquisas Aggeu Magalhães.; 4 Joint Professor, Discipline of Otorhinolaryngology, Federal University of Pernambuco. Head of the service of Otorhinolaryngology, Hospital das Clínicas, Federal University of Pernambuco.; 5 Histotechnician, Master studies, Department of Pathology, Hospital das Clínicas, Federal University of Pernambuco.

**Keywords:** meningites, cochlear implant, deafness, children

## Abstract

Myringosclerosis is characterized by hyaline changes of the lamina propria of the tympanic membrane. Experimental studies have used otomicroscopy or histology to evaluate myringosclerosis in animals, but they do not correlate precisely these two methods. **Aim:** The present study evaluates the accuracy of otomicroscopy in the diagnosis of myringosclerosis in rats. **Study design:** experimental. **Material and Method:** Myringosclerosis was induced by transtympanic inoculation of *Streptococcus pneumoniae* in 25 Wistar rats, which were examined weekly through otomicroscopy and sacrificed after eight weeks for histologic study of their tympanic membranes. **Results:** From the comparison of the otomicroscopic data in relation to the histologic findings, we could observe sensibility of 80% and specificity of 75% for the otomicroscopy. **Conclusion:** Considering the results in this study, otomicroscopy did not represent a good method to evaluate myringosclerosis in this experimental model.

## INTRODUCTION

Tympanosclerosis is the term used to describe a histological abnormality that occurs in the middle ear mucosa (ME) or mastoid characterized by hyalinization, which may progress to calcification or ossification ^1^. It was described for the first time by Von Tröltsch, in 1873, who reported a pathological process characterized by ME mucosa rigidity ^2^. Later, Zöllner; Beck, in 1955, introduced the term tympanosclerosis.^3^

It is the final stage of a process originated from acute, chronic or recurrent inflammation of ME lamina propria, characterized by progressive infiltrate of fibroblasts, causing an increase in collagen, which originates poor formation of cells and blood vessels, followed by hyaline degeneration and in some occasions, calcium deposit 1, 4, 5, 6, 7, which may lead to formation of cartilage or bone ^8^. In some cases there may be functional damage for the tympanic-ossicle unit, causing conductive hearing loss, which affects surgical decisions during tympanoplasties.

When the tympanosclerosis affects the tympanic membrane (TM), it may be diagnosed with otoscopy, showing an aspect of white plaques and variable extension, named myringosclerosis .^9^

Experimental models have been used to induce tympanosclerosis from the beginning of the 1970^10^, ^11^, ^12^, ^13^, ^14^, ^15^, ^16^, ^17^, ^18^, ^19^, ^20^, ^21^, ^22^, most of them performed in rats. These models used to be studied based on otoscopy, otomicroscopy and histology, with varied purposes, such as determination of etiological factors, study of etiopathogenesis, therapeutic drug action, etc. The advantages of the otomicroscopic TM assessment in relation to the histological study are easy and quick execution, low costs and no need to sacrifice the animal. However, we do not know to what extent the otomicroscopic findings may be reliable to identify myringosclerosis. The lack of correlation between otomicroscopy and histological confirmation, for obvious reasons, may lead to inappropriate conclusions in studies that involve induction of myringosclerosis in experimental models. No literature research study has made the appropriate correlation of findings and animals. The present study, therefore, aims at assessing the level of correlation between otomicroscopic aspect and histological TM findings in rats that suffered induction of myringosclerosis.

## MATERIAL AND METHOD

The present study was performed at the Nucleus of Experimental Surgery, Federal University of Pernambuco, in which the study with the animal model was performed, from preparation, inoculation, otomicroscopy, and sacrifice of the animals for the histological study; the preparation and analysis of slides were performed at the Department of Clinical Analysis, UFPE and Department of Cellular Biology, Centro de Pesquisa Aggeu Magalhães.

Twenty-five (25) Wistar rats, healthy, weighting between 200-400g, young adults, were maintained in the Nucleus of Experimental Surgery, in individual cages with pine wood saw dust flooring, artificial lighting, room temperature and feed. The rats were anesthetized with chloral hydrate at 10% by intra-peritoneal injections after which they were placed over the surgical bench, left lateral position, so that the examiner could explore the right ear.

Under otomicroscopy, using surgical microscope brand D.F. Vasconcelos, model MC-M31, we inoculated in the middle ear of each animal, via transtympanic access, 0.1ml of solution containing 10^7^ colony-forming units (CFU) of *Streptococcus pneumoniae type 3,* after which the animals were sent back to their respective cages.

For a period of 8 weeks, two examiners performed otomicroscopies every 15 days in all rats under general anesthesia. After 1 week, all rats presented signs of acute otitis media and in the last otomicroscopy, tympanic membranes were photographed with digital camera brand Sony, model MVC-FD73, coupled to the division system of the microscope rays. In addition to photos of membranes we marked down and drew the abnormalities found. After it, the rats were sacrificed with a lethal dose of chloral hydrate.

After the sacrifice, tympanic bullae were removed, and we isolated the TM together with the external auditory canal. The pieces were fixed in formaldehyde at 10% and decalcified in nitric acid at 7.5%. In the post-fixation process, bullae were placed in a hot over for 40 minutes in alcohol and 20 minutes in alcohol-xylol and later they were placed in xylol outside the hot oven, and then they were dehydrated, mounted in paraffin bloc (50 minutes). The material was sectioned at axial sections of 4µm thickness, with intervals of 50µm, encompassing the whole TM, and stained with hematoxylin-eosin and then examined under optical microscopy.

**Figure 1:**
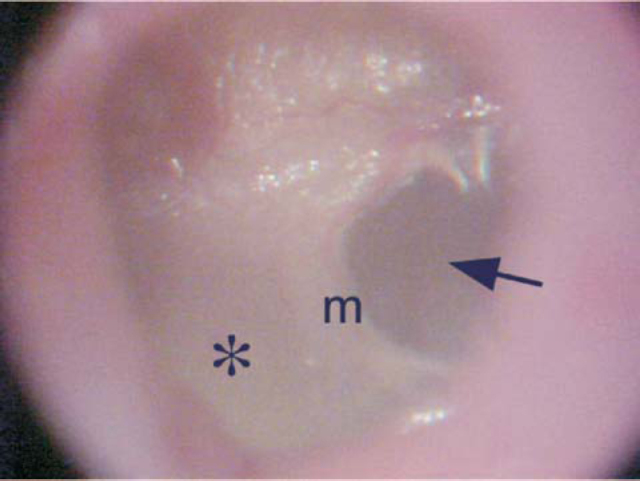
Otomicroscopy showing marked opacification of the posterior portion of the tympanic membrane (*), showing the anterior half with normal transparency (arrow). m – malleus manubrium.

**Figure 2:**
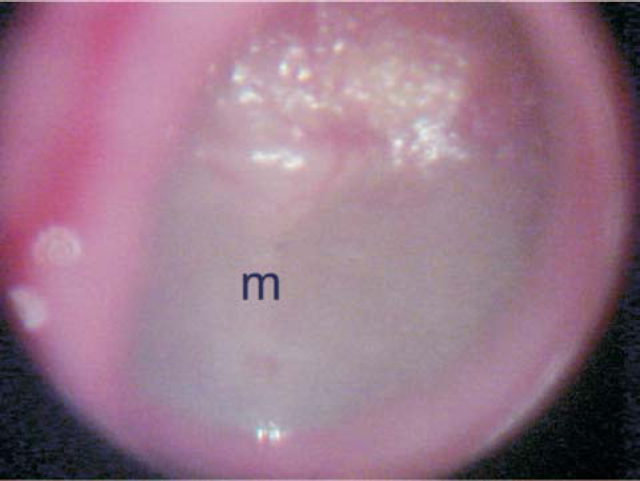
Otomicroscopy showing marked diffuse opacification of the tympanic membrane. m – malleus manubrium.

At otomicroscopy, we defined the following aspects:

**a.** **Normal TM** – Defined as thin and transparent tympanic membrane;
**b.** **Mild opacification** – Defined as mild loss of transparence of TM, which may occur diffusely or in isolated areas;
**c.** **Marked opacification** – Defined as the white plaque, with total loss of transparency and marked TM thickness, which occurs diffusely or in isolated areas (Figures 1 and 2).

**Figure 3:**
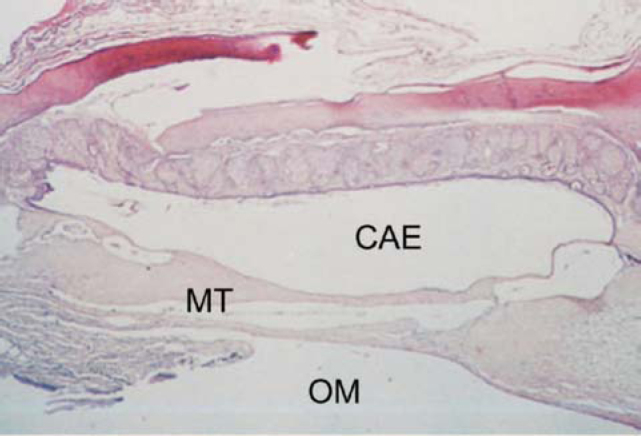
Axial section stained with HE, 10 X, of the tympanic membrane (MT) with tympanosclerosis phase 3. CAE – external auditory canal; OM – middle ear.

**Figure 4:**
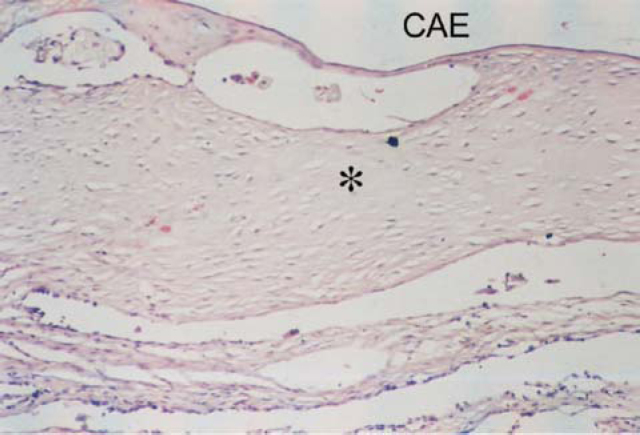
Detail of Figure 1: Note the sparse fibroblasts, involved by hyaline collagen (*). CAE – external auditory canal.
**e.** **Phase 5** – calcification, characterized by calcium and phosphorus deposits, giving the collagen matrix an aspect similar to that of cartilage or bone tissue (Figures 5 and 6).

At the histological examination, we characterized the stages of the inflammatory process that peaked as myringosclerosis, defined into 5 phases:

**a.** **Phase 1** – exsudation, characterized by marked polymorphonuclear infiltrate;
**b.** **Phase 2** – granulation, characterized by marked neovascularization and presence of elements of the macrophage mononuclear system (fibroblasts, lymphocytes, macrophages);
**c.** **Phase 3** – fibrosis, characterized by major proliferation of fibroblasts and formation of collagen fibers, reduction of vascularization;
**d.** **Phase 4** – hyalinization, characterized by reduction in number of fibroblasts, which are replaced by collagen fibers, which fuse forming plaques (Figures 3 and 4);

**Figure 5:**
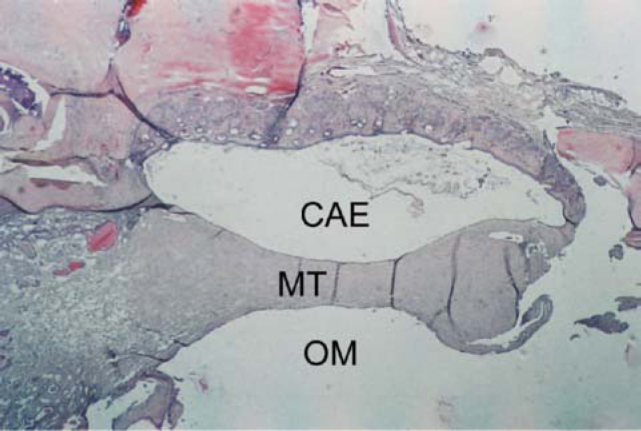
Axial section stained with HE, 10 X, of tympanic membrane (MT) with tympanosclerosis phase 4. CAE – external auditory canal.; OM – middle ear.

**Figure 6:**
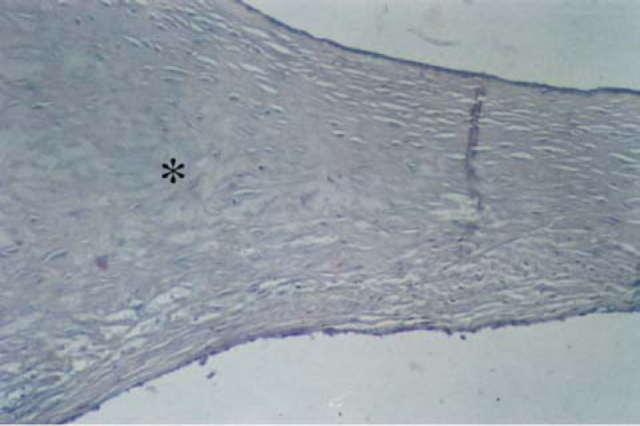
Detail of Figure 3. We can observe the typical aspect of bone metaplasia (*) on the medium layers of the tympanic membrane.

Given that up to Phase 3 the abnormalities are seen as reversible, we considered as myringosclerosis only the material that presented at least one section with aspect of phases 4 or 5, considering that the main histological characteristics of tympanosclerosis is the presence of hyalinization, with our without calcification.

Data were described under the form of absolute and relative frequencies, depending on the studied categories. To compare the otoscopic and histological diagnoses, McNemar test was employed given that they were the same subjects assessed in both occasions.

The assessment of agreement between diagnosis with the referred techniques was made by determining Kappa indexes attributed to the following scores for level of agreement 0-20 (weak), 20-40 (poor), 40-60 (fair), 60-80 (good), 80-100 (excellent).

The present study complied with the principles of experimental ethics and animal protection laws, according to the norms in force in Brazil, especially Law 9.605, art. 32 and Decree 3.179, art. 17, dated 21/09/1999, that addresses the use of animals in scientific studies. It was approved by Ethics Committee on Animal Experiment, Center of Biological Sciences, Federal University of Pernambuco (CEEA-UFPE), including the agreement that sacrifice of these animals in the present study was justified because of the fact that there were no alternative resources to perform the same scientific procedure.

## RESULTS

Otomicroscopic exam was considered normal in four examined ears (16%). Mild opacification was found in 13 ears (52%) and marked opacification (white plaques) in 8 (32%), as we can seen in Table 1. Histological findings were: normal histology in four animals (16%); up to phase 3 in 15 ears (60%) and beyond phase 3 in 6 (24%) membranes (Table 2).

**Table 1:** Distribution of animals based on otoscopy.

Diagnosis (Classification)	n	%
Normal	4	16.0
Mild	13	52.0
Marked	8	32.0

Total	25	100.0

**Table 2:** Distribution of animals based on histology.

Diagnosis (Classification)	n	%
Normal	4	16.0
≤ 3	15	60.0
> 3	6	24.0

Total	25	100.0

By comparing both methods, we can see in Table 3, that among the 8 rats that presented marked opacification at otomicroscopy, four presented phase 4 or 5, whereas 4 presented up to phase 3; there were no normal cases. Among the 13 ears that had mild opacification, only 2 had histological findings beyond phase 3, 10 had phase 3 histology and one had normal histology. Finally, out of the four rats that had normal otomicroscopy, one had phase 1 histological findings and the others had normal mucosa histology (p=0.290).

**Table 3:** Agreement between otoscopic and histologic diagnosis.

		Histologic		
		> 3	≤ 3	Normal
Otoscopic	Marked	4	4	0
	Mild	2	10	1
	Normal	0	1	3

	Total	6	15	4

Comparison: McNemar test: p = 0.290

Kappa index 0.46

Table 4 simplifies these data by gathering normal and mild opacification otomicroscopic findings in one single group, considered negative for myringosclerosis, and also normal and phase ≤*3* in a single group, also considered to be negative. From the analysis, we learned that among the rats with positive otomicroscopy there were four with positive histology and five with negative histology. Out of the ears considered to be negative at otomicroscopy, one was positive and fifteen were negative at the histology exam, which revealed sensitivity of 80% and specificity of 75% in findings of otomicroscopic exam and histological exam.

**Table 4:** Accuracy

		Histologic	
		> 3	< 3 ou Normal
Otoscopic	Marked	4	5
	Mild/Normal	1	15

Measurements of association and confidence interval of 95%

Sensitivity: 80.0% (29.9 – 98.9)

Specificity: 75.0% (50.6 – 90.4)

Positive predictive value: 44.4% (15.3 – 77.3)

Negative predictive value: 93.8% (67.7 – 99.8)

## DISCUSSION

Tympanosclerosis has been the reason for many literature studies. It is a pathological process that is quite prevalent in our country because it is frequently detected in acute infections, especially chronic ME mucosa infections 1, 9. Despite the fact that they had been well defined in their histological characteristics 1, 4, 5, 6, 7, 8, etiology and pathogenesis are not completely explained and better understanding of these aspects may bring future possibilities of preventive treatment.

For this reason, experimental studies are extremely important to try to define all pathological steps of the development of tympanosclerosis, histochemical factors and molecular affections also involved. Some studies may use otomicroscopic assessment or histology to study the incidence and dynamics of the formation of tympanosclerosis in animals in view of a possible etiological factor^12^, ^14^, ^18^, ^19^, ^20^. Other studies tried to understand the etiopathogenic dynamics of tympanosclerosis, characterized by histological and ultrastructural affections.^15^, ^17^, ^22^ There are many studies that aimed at finding some beneficial effect of substances that may prevent progression of tympanosclerotic disease.^13^, ^16^, ^17^, ^21^ Finally, there are studies directed only to presentation or discussion with an experimental model.^10^, ^14^

**Table:** Table describing the otomicroscopic and histological findings in each ear.

EARS	OTOMICROSCOPY	HISTOLOGY
A1OD	OL	N
A2OD	OI	PHASE 4
A4OD	OL	PHASE 3
A6OD	OI	PHASE 3
A11OD	OL	PHASE 3
A140D	OL	PHASE 3
A15OD	OL	PHASE 4
B1OD	OI	PHASE 3
B3OD	N	PHASE N
B4OD	OI	PHASE 3
B6OD	OI	PHASE 4
B9OD	OL	PHASE 2
B10OD	N	N
B12OD	OL	PHASE 3
B13OD	OL	PHASE 3
B16OD	OL	PHASE 3
C2OD	OL	PHASE 4
C3OD	OI	PHASE 4
C5OD	OI	PHASE 3
C6OD	OL	PHASE 3
C10OD	N	N
C11OD	N	PHASE 3
C12OD	OL	PHASE 1
C16OD	OL	PHASE 2
C18OD	OI	PHASE 4

Key: OL - Mild Opacification; N - Normal; OI - Marked Opacification

Many experimental studies use otomicroscopy as the assessment method for tympanosclerotic process 10, 11, 14, 16, 18, 22, but none of them tried to precisely correlate the affections found in the assessment and histology findings, meaning that there is no single study that shows reliability of images seen through otomicroscopy, that is, exam accuracy.

Otomicroscopy is easily performed under general anesthesia, not causing any suffering to the animal and not requiring sacrifice. Evidently, mere observation of otomicroscopic affection of the TM is not capable of providing data about histostructural or histochemical affections present in the tympanosclerosis process, which are essential aspects for the formulation of many different etiopathogenic or pathophysiological hypotheses. It is also clear that otomicroscopy allows only identification of tympanosclerosis seen in the TM (myringosclerosis). It has been demonstrated that tympanosclerosis may occur only inside the middle ear, sparing the TM 1, 9. Thus, studies performed using otomicroscopy as an isolated method of assessment of tympanosclerosis may occasionally underestimate the incidence of tympanosclerosis because it does not identify plaques that do not involve the TM.

However, to those studies directed to introduction of new experimental models, as well as to those directed to defining comparative case-control analyses, otomicroscopy can be extremely useful. In chronological studies of tympanosclerosis progression, isolated otomicroscopy may satisfy methodological needs. However, for it to happen, it is essential that images observed by otoscopic exam of TM have good specificity, sensitivity and accuracy, running the obvious risk of leading to misinterpretation of presence or absence of myringosclerosis.

Myringosclerosis is in general an easy to identify affection at otoscopy as white plaques on the TM ^9^, completely opaque, that clearly contrast with normal areas, which can have very expressive transparency. However, otoscopic images of the areas with intermediate aspect on the TM are very common, normally accepted as normal, and the literature does not bring any histological study in these areas. In rats, especially owing to reduced TM size, aspects my get confused. Total opacification of the TM may represent acute or subacute edematous process or repairing process, with or without tympanosclerosis.

In the present study, however, we tried to correlate otomicroscopic findings with the affections found in the histological assessment of the TM. We used rats as experimental model because they have many practical advantages and similar anatomical and pathological conditions to human ME ^14^, and also because it is the most commonly used animal for this purpose in the literature.

The technique of tympanosclerosis induction using transtympanic inoculation of ME with type 3 *Streptococcus pneumoniae* was also chosen owing to its simplicity, as well as the anesthetic method. We performed otomicroscopy every 15 days for a period of 8 weeks because many aspects of the TM may interfere in the progression of the inflammatory process. Eight weeks were selected for the sacrifice because in most studies, histological affections compatible with tympanosclerosis may be seen after this period.

The histological method used, optical microscopy with hematoxylin-eosin, enables easy identification of all phases of the inflammatory process that peaks with tympanosclerosis. It is defined in the literature as hyaline degeneration that occurs in the collagen matrix that is formed during the repairing process, which can deposit calcium and phosphorus crystals.^1^, ^5^, ^6^, ^7^ However, these deposits are not always present and the tympanosclerotic process may be characterized basically by hyalinization of neoformed collagen tissue of lamina propria and we considered as positive for myringosclerosis if there were phases 4 or 5 of the studied tissue.

Otomicroscopic findings, despite being widely varied, may be classified into 3 groups: normal (when transparency is preserved, equal to the other ear); mild opacification, and marked opacification (white plaques).

McNemar test was used to check whether there were changes in the two diagnostic methods. We had one case with result of p=0.290, indicating that there were no significant changes, that is, there was association between the two methods. Kappa index is also an agreement index and it was used to express test reliability. The result obtained (0.46) indicates regular agreement between the diagnostic methods. It occurred probably owing to wide variability of findings (Table 3). Considering as myringosclerosis the presence of marked opacification (Plaque) in the otomicroscopic exam and phases 4 or 5 (hyalinization or metaplasia) in the histological exam and absence of tympanosclerosis, normal exam or mild opacification in the otomicroscopic exam and up to phase 3 (fibrosis) in the histological exams, we tried to study the accuracy of results obtained in this study (see Table 4). Through the test, we reached sensitivity of 80% and specificity of 75%. However, the confidence interval was wide, owing to the small number of animals in the subgroup with positive otomicroscopy. Among those with normal otomicroscopy, there was clear agreement with histological findings. It demonstrates that otomicroscopy is a good method to predict the proportion of true negative results, but findings did not enable us to state the good correlation between findings of myringosclerosis at otomicroscopy and histology findings.

## CONCLUSION

Based on the results of the present study, otomicroscopy did not prove to be a good method to assess myringosclerosis in this experimental model.
